# The Association Between Effectiveness of Tinnitus Intervention and Cognitive Function—A Systematic Review

**DOI:** 10.3389/fpsyg.2020.553449

**Published:** 2021-01-06

**Authors:** Tianxiang Lan, Zuwei Cao, Fei Zhao, Nick Perham

**Affiliations:** ^1^Centre for Speech and Language Therapy and Hearing Sciences, Cardiff School of Sport and Health Sciences, Cardiff Metropolitan University, Cardiff, United Kingdom; ^2^Center for Rehabilitative Auditory Research, Guizhou Provincial People's Hospital, Guiyang, China; ^3^Department of Hearing and Speech Science, Xinhua College, Sun Yat-sen University, Guangzhou, China; ^4^Department of Applied Psychology, Cardiff School of Sport and Health Sciences, Cardiff Metropolitan University, Cardiff, United Kingdom

**Keywords:** tinnitus, cognition, tinnitus sound therapy, intervention, cognitive behavioral therapy

## Abstract

Tinnitus refers to the perception of sound in the absence of an external stimulus. This can be problematic and can lead to health problems in some sufferers, including effects on cognitive functions such as attention and memory. Although several studies have examined the effectiveness of tinnitus interventions, e.g., cognitive behavioral therapy and sound therapy, it is still unclear as to the overall quality and limitations of these studies and whether their results could be generalized. Clarification is also needed as to whether poor cognitive function will lead to a less favorable intervention outcome in tinnitus patients. The present systematic review was therefore designed to critically appraise and synthesize findings from randomized controlled trials (RCTs) of tinnitus intervention and its effects on cognition. The methodology followed the Preferred Reporting Items for Systematic Reviews and Meta-Analysis (PRISMA). Medline (PubMed), Embase, and PsycINFO were searched. Only RCTs that compared the effectiveness of a tinnitus intervention and a measure of cognitive function in adult participants with tinnitus were included. A total of 8 studies involving 610 participants tested using 11 cognitive function assessment tools (e.g., Stroop Color and Word Test and Visual Continuous Performance Task) and 5 tinnitus intervention outcome measurements (e.g., Tinnitus Handicap Inventory and Tinnitus Questionnaire) were included and analyzed. The outcomes of the review suggest that tinnitus intervention not only facilitates tinnitus management but also improves cognitive functions. It is likely that cognition and emotion play an important role in a patient's adjustment to tinnitus. Whether cognition can predict treatment outcomes is unclear due to insufficient evidence. Future research is needed using a standardized assessment protocol focusing on the effect of sound-based interventions on tinnitus severity and cognitive functions. Studies on whether cognitive function measurement can be used as a predictor for the effectiveness of tinnitus therapy are also needed.

## Introduction

Subjective tinnitus refers to the perception of sound without the presence of an external stimulus (De Ridder et al., 2014). It is commonly experienced, and almost all adults have had some form of temporary tinnitus at some point, but often without significant annoyance or discomfort (Tyler et al., 2004). Tinnitus can however be highly problematic if symptoms persist and cause health problems, such as insomnia, hearing difficulties, and psychological problems (Tyler et al., 2004). Others with chronic and severe tinnitus can complain of anxiety, depression, and cognitive function-related issues, such as difficulty concentrating (Tyler and Baker, 1983; Andersson et al., 1999). Prevalence studies indicate that persistent tinnitus symptoms affect 10–17% of adults in the United States of America and Europe, with 2–7% of these seeking medical advice and treatment (Davis and El Refaie, 2000; Holmes and Padgham, 2009; Langguth et al., 2013).

In general, cognitive functions are the mental processes that enable us to receive, select, store, transform, develop, and recover information from external stimuli (Stevens and Bavelier, 2012; Clarke et al., 2018). They are associated with concentration, attention, and use of memory, which closely correlate with daily activities and life experience involving communication, perception, thoughts, and emotions (Andersson and McKenna, 2006). Good cognitive function is essential to enable people to recognize and understand the meaning of language and their environment.

Andersson and McKenna (2006) suggest that cognitive function plays an important role in the pathway from tinnitus perception to annoyance. In the study by Hallam et al. (2004), five cognitive measures were conducted under single and dual-task conditions to test sustained attention, reaction time, verbal fluency, and immediate and delayed memory. Patients with tinnitus had significantly slower responses than participants without tinnitus on the variable fore-period reaction time task under dual-task conditions. It indicated that that tinnitus sufferers had to pay more efforts to maintain attention on daily activities in comparison to people without disturbance caused by tinnitus.

Further evidence also suggested that patients with tinnitus had poor performance relevant to attention management, particularly in task-based behavior, revealing a slow response and reduced accuracy on cognitive performance tests for attention and working memory (Rossiter et al., 2006; Pierce et al., 2012; Jackson et al., 2014; Cardon et al., 2019). For example, the patients with tinnitus had significantly lower scores or longer response times on cognitive performance tests (i.e., Stroop Color and Word test assessing the ability to inhibit cognitive interference) in comparison to normal healthy control participants without tinnitus (Jackson et al., 2014; Cardon et al., 2019). Although an association between self-reported cognitive dysfunction and poor cognitive performance in patients with tinnitus has been suggested (McKenna et al., 1995; Hallam et al., 2004), Mohamad et al. (2016) argue that any correlation between self-reported failures in cognition and cognitive performance is unclear due to a lack of consistent findings.

Additionally, psychological issues such as depression and anxiety in tinnitus patients could potentially contribute to their poor cognitive performance (Tegg-Quinn et al., 2016), with a certain level of selection bias and other confounding factors likely to influence analysis of tinnitus and cognitive function (Mohamad et al., 2016). For example, using a self-reported assessment of the Cognitive Failure Questionnaire (CFQ), Hallam et al. (2004) revealed that more cognitive difficulties reported in the CFQ significantly correlated with a higher degree of anxiety in patients with tinnitus. It implies that some cognitive problems are influenced by the psychological issues associated with tinnitus. Therefore, several recent review studies suggest that further investigations on the impact of tinnitus on cognitive function are required (Mohamad et al., 2016; Tegg-Quinn et al., 2016; Trevis et al., 2018).

These reviews did not, however, incorporate studies on tinnitus interventions and currently no review has been conducted relating to the association between tinnitus interventions and cognitive function. Several interventions such as sound therapy and cognitive behavioral therapy (CBT) have been implemented clinically to manage tinnitus-related symptoms, with effectiveness demonstrated in numerous studies (Hesser et al., 2011; Pienkowski, 2019). CBT is currently recognized as one of the most effective interventions for tinnitus management (Hesser et al., 2011; Fuller et al., 2020). A very recent systematic review shows that CBT has better outcomes in terms of reducing the detrimental impact of tinnitus on quality of life in comparison to audiological interventions such as sound therapy and tinnitus retraining therapy (Fuller et al., 2020).

The importance of cognitive function and emotion has been highlighted in a review by Hesser et al. (2011), who conclude that CBT focuses on changes to a patient's emotional and/or behavioral response, making it an effective tool to alleviate tinnitus-related distress. This also implies that cognitive function might be crucial in the management of tinnitus. Although several studies have investigated the association between tinnitus therapy and cognitive function, the evidence remains limited and controversial. For example, Kallogjeri et al. (2017) suggested that a computer-based brain fitness program was associated with self-reported changes in attention, memory, and perception of tinnitus, together with self-reported improvement in tinnitus perception, but no significant changes in the cognitive performance tasks of memory and sustained and selective attention between patients with tinnitus and controls. In contrast, Krick et al. (2017) showed alleviation in the severity of tinnitus and improvement in the cognitive performance tasks of memory and sustained and selective attention using music therapy. Such discrepancies are likely due to heterogeneity in study design, i.e., these studies used different types of outcome measurements, interventions, and cognitive performance tasks, and thus making a comparison of these studies challenging.

Furthermore, although Kröner-Herwig et al. (2006) attempted to investigate cognitive characteristics as a predictor of the effectiveness of tinnitus intervention, there was no significant correlation between cognitive performance and intervention outcomes in patients with tinnitus assessed using Tinnitus-Dysfunctional-Cognitions Questionnaire and Tinnitus Questionnaire, respectively (*r* = −0.21, *p* > 0.05). However, Conrad et al. (2015) found a negative correlation between a subscale of Tinnitus Cognitions Scale and Tinnitus Handicap Inventory post CBT (*r* = −0.36, *p* = 0.045). The inconsistent findings are likely due to differences in the study design, such as assessment tools for measuring cognition function and tinnitus-related questionnaires. Therefore, whether a poor performance in cognitive tasks would lead to a less favorable outcome in patients with tinnitus remains unclear. Because randomized controlled trials (RCTs) have an important advantage in terms of minimizing differences in characteristics of the groups that may influence the outcome, for the purpose of providing strong evidence for interpreting and critically evaluating clinical research data, the present review aimed to critically appraise and synthesize findings obtained from the RCT studies, which would address the efficacy of tinnitus intervention on tinnitus and cognitive function in adult patients with tinnitus, and thus facilitate to translate research data into clinical practice. In addition, whether the cognitive function can be used as a predictor for interventional outcomes was also examined.

## Methods

The research questions were raised and formulated according to the PICOS principle, i.e., *Patients* (patients with tinnitus), *Intervention* (specific tinnitus management, e.g., sound therapy and cognitive behavioral therapy), *Comparison* (with and without interventions), *Outcomes* (measurements in tinnitus severity and cognitive performance), and *Study Design* (RCTs) (Siadaty et al., 2007). The methodology of this review follows the checklist of Preferred Reporting Items for Systematic Reviews and Meta-analysis (PRISMA).

### Information Sources and Search Strategy

Studies were identified through the following electronic database: Medline (PubMed), Embase, and PsycINFO. The search was conducted in March 2020. In addition to searching databases, hand searching of reference lists from identified studies was conducted. The keywords used to search were first identified by reviews on tinnitus and cognitive function. Then, Medical Subject Headings (MeSH) were used to confirm the final search terms.

The following search terms and strategy were used to search the databases:

- #1 Search: (Tinnitus[MeSH Terms]) OR (Subjective Tinnitus) (Hits: 35,952)
- #2 Search: (((((Cognition[MeSH Terms]) OR (Cognitive Function)) OR (Function, Cognitive)) OR (Attention)) OR (Concentration)) OR (Memory) (Hits: 6,382,500)
- #3 Search: ((((((((tinnitus management) OR (tinnitus therapy)) OR (tinnitus intervention)) OR (tinnitus sound therapy)) OR (tinnitus music therapy)) OR (tinnitus masking)) OR (cognitive behavioral therapy)) OR (cognitive psychotherapy)) OR (cognition therapy) (Hits: 114,344)
- #4 Search: ((#1) AND (#2)) AND (#3) (Hits: 668)
- #5 Search: ((#1) AND (#2)) AND (#3) Filters: Randomized Controlled Trial (Hits: 98).

### Inclusion Criteria

Randomized control trials studying the impact of tinnitus interventions on cognition or whether cognition is a predictor of intervention outcomes were considered. Only published, English, and peer-reviewed journal articles were considered to be included in this review. There was no restriction on the date of publication.

#### Participants

Studies including participants over 18 years of age with subjective tinnitus were considered. There was no restriction on tinnitus duration and severity.

#### Interventions

This review was limited to RCTs on tinnitus managements such as sound therapy and cognitive behavioral therapy, but studies on the outcomes of tinnitus intervention using medications or surgical operation alone were excluded.

#### Outcome Measurements

Self-reported measurements of cognitive function or behavioral measurements of cognitive function.

### Study Selection

The title and abstract of studies retrieved from databases were first screened by the first author. Repeated records and studies that did not meet the criteria were excluded. A full-text screening against the inclusion criteria was then conducted on the remaining records. Discrepancies were resolved by FZ OR NP.

### Risk of Bias Assessment

To evaluate the methodological quality of included studies, version 2 of the Cochrane Risk-Of-Bias tool for randomized trials was used to assess the adequacy of randomization, concealment of allocation processes, blinding of participants and trial personnel, the extent of missing outcomes, and selective outcome reports. In addition, RevMan 5.3 software was used to plot a figure showing the risk of bias.

### Data Extraction

Descriptive data extraction was conducted by the first and second authors (TL and ZC) with use of the agreed data collection form. Data extracted included the type of study design, participant age and gender, description of tinnitus (severity and duration), and outcome measurements (types of cognitive evaluation). In addition, bias assessment was undertaken to show the overall quality of the included studies. Any disagreements were arbitrated by one of the other authors (FZ and NP) after discussion.

## Results

### Study Selection Procedure and Outcome

**Figure 1:**
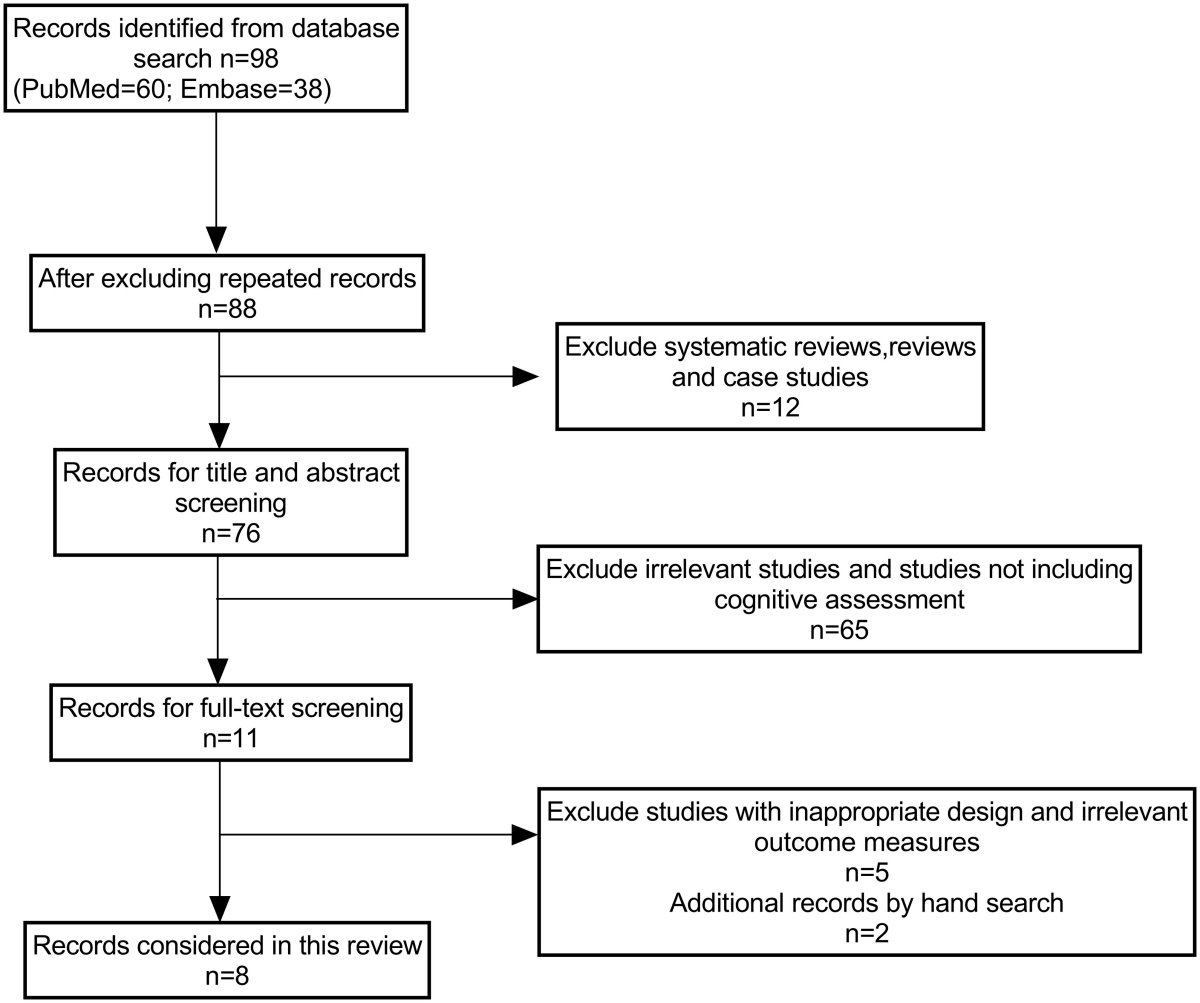
A flow chart illustrating the study selection process.

Ninety-eight studies were identified, 62 of which were identified from the PubMed (Medline), and 36 were identified through Embase and PsycINFO. A further two were found by manual references searching in the included studies because the terms used in the search strategy were not included in the title or abstract or the keyword list of these two studies (Hiller and Haerkötter, 2005; Kröner-Herwig et al., 2006). After adjusting for repeat records, 89 remained. Of these, 78 studies were excluded after screening the title and abstract, as they did not meet the inclusion criteria because these studies used medical interventions for tinnitus management (e.g., medications or transcranial magnetic stimulation therapy). Following the full-text screening, 3 out of 11 studies were excluded because these studies measured neural activities associated with cognitive function in responding to tinnitus intervention. Finally, eight randomized controlled studies were included in this review. A flow diagram of the selection processes is shown in Figure 1.

### Characteristics of Included Studies

As shown in Table 1, all eight included studies were designed as randomized controlled trials; however only one was conducted using a double-blind design (Krings et al., 2015). A total of 335 male and 275 female participants were included in the eight studies. All participants suffered chronic tinnitus defined as minimum 6 months in duration. The study by Krick et al. (2017) included participants with recent-onset tinnitus (i.e., persistent tinnitus <6 months) as a control group. No significant differences in the baseline characteristics of participants were suggested in all studies except the study by Hesser et al. (2009). This study showed the control group was significantly younger than the study group (control group vs. study group: 40.6 vs. 52.2, *p* = 0.016).

**Table 1:** Characteristics and important information of studies included in this review.

**Author**	**Aim**	**Demographic data and tinnitus characteristics of the participants**		**Intervention and measurements**		**Key relevant results**
		***Sample size***	***Tinnitus* characteristic**	***Intervention***	***Outcome measurement***	
Conrad et al. (2015)	• Effect of CBT on cognitions • Cognitions as predictor of therapy outcome	**Total: 128 participantsICBT group:** 41 participants Gender: M = 25/F = 16 Age: 51.32 ± 9.78 years **GCBT group:** 43 participants Gender: M = 24/F = 19 Age: 50.23 ± 13.13 years **DF group:** 44 participants Gender: M = 28/F = 16 Age: 52.09 ± 8.99 years	**ICBT group:** Duration: 9.23 ± 7.88 years THI score: 40.34 ± 17.64 **GCBT group:** Duration: 8.35 ± 6.85 years THI score: 44.33 ± 19.17 **DF group:** Duration: 7.88 ± 7.91 years THI score: 40.23 ± 20.54	**ICBT group:** Internet Cognitive Behavioral Therapy **GCBT group:** Cognitive Behavioral Group Therapy **DF group:** Online Discussion Forum	• Self-reported measure • Tinnitus Cognitions Scale	• Improvement in Tinnitus Cognitions Scale and THI were suggested in both ICBT and GCBT. • Negative correlation was found between subscale of the T-Cog and outcome of ICBT.
Hesser et al. (2009)	• Effects of controlled background sounds on cognitive performance and tinnitus intrusiveness	**Total: 35 participantsStudy group:** 18 participants Gender: M = 11/F = 7 Age: 52.2 ± 10.8 years **Control group:** 17 participants Gender: M = 10/F = 7 Age: 40.6 ± 16.0 years	**Study group:** Duration: 9.7 ± 7.7 years THI score: 48.4 ± 21.7 TRQ score: 36.4 ± 21.7 **Control group:** Duration: 6.2 ± 8.5 years THI score: 46.3 ± 24.4 TRQ score: 33.2 ± 27.1	**Study group:** A background sound and loudness level participant preferred **Control group:** Background sound and loudness level were determined	• The Digit-Symbol Substitution Test	• Compared with no control condition, group with controlled background sounds showed increased tinnitus interference and slower rates of improvement on cognitive performance.
Hoare et al. (2014)	• Effects of game based FDT on intrinsic motivation. • Effects of game based FDT on cognitions	**Total: 60 participantsGroup A:** 20 participants Gender: M = 12/F = 8 Age: 60.2 ± 12.5 years **Group B:** 20 participants Gender: M = 12/F = 8 Age: 57.8 ± 14.0 years **Group C:** 20 participants Gender: M = 10/F = 10 Age: 60.6 ± 11.4 years	**Group A:** Duration: 12.6 ± 11.9 years THQ score: 906 ± 485 **Group B:** Duration: 10 ± 9.7 years THQ score: 937 ± 452 **Group C:** Duration: 11.4 ± 11.2 years THQ score: 1040 ± 440	**Group A:** Conventional FDT **Group B:** Interactive Game-Based FDT (**Treasure Hunter)** **Group C:** Interactive Game-Based FDT (**Submarine**)	• Test of Everyday Attention	• No significant intervention effects on sustained attention were observed. • Changes in tinnitus severity were not significant in all groups.
Hiller and Haerkötter (2005)	• Whether sound stimulation enhances the effects of CBT	**Total: 124 participantsTinnitus Education: 64 participants** **Study group**: 31 participants Gender: M = 16/F = 15 Age: 52.5 ± 15.3 years **Control group**: 33 participants Gender: M = 20/F = 13) Age: 45.2 ± 14.1 years	**Tinnitus Education Study group:** Duration: none reported TQ score: 26.9 ± 10.7 **Control group:** Duration: none reported TQ score: 24.4 ± 9.0	**Tinnitus Education** **Study group:** Tinnitus Education with Noise Generator **Control group:** Tinnitus Education without Noise Generator	• Tinnitus Cognitions Scale	• All groups improved, however not significant, on tinnitus-related distress, dysfunctional cognitions, depression, psychosocial functioning. • Sound stimulation does not further improve the effects of CBT.
		**CBT: 60 participants** **Study group**: 31 participants Gender: M = 21/F = 10 Age: 51.0 ± 13.2 years **Control group**: 29 participants Gender: M = 12/F = 17 Age: 51.4 ± 10.9 years	**CBT** **Study group:** Duration: none reported TQ score: 53.4 ± 12.4 **Control group:** Duration: none reported TQ score: 48.8 ± 12.8	**CBT** **Study group:** CBT with Noise Generator **Control group:** CBT without Noise Generator		
Kallogjeri et al. (2017)	• Effect of the BFP-T on tinnitus and cognitive functions.	**Total: 60 participants** **Study group:** 20 participants Gender: M = 13/F = 7 Age: 56 (35–64) **median (range)** **Control group:** 20 participants Gender: M = 14/F = 6) Age: 52 (24–64) **median (range)** **Health Control:** 20 participants Gender: M = 13/F = 7 Age: 50 (30–64) **median (range)**	**Study group:** Duration: 3.8(0.5–35.0) THI score: 37(14–80) TFI score: 37.8(20.4–62.8) **Control group:** Duration: 9.0(0.5–30.0) THI score: 36(12–70) TFI score: 43(6.4–80.4) **Health Control:** Duration: NA THI score: NA TFI score: NA **In median (range)**	**Study group:** BFP-Tinnitus **Control group:** No intervention **Health Control:** No intervention	• The California Verbal Learning Test • Wechsler Memory Scale—Fourth Edition • Conners Continuous Performance Test • Cognitive Failure Questionnaire	• The findings suggest that the computer-based cognitive training program is associated with self-reported changes in attention, memory and tinnitus perception.
Krick et al. (2017)	• Effects of tinnitus duration on visual attention. • Effects of HNMT on the visual attention network	**Total: 113 participants** **Chronic Tinnitus**: 33 participants Gender: M = 21/F = 12 Age: 47.6 ± 10.4 years **Recent Tinnitus:** 45 participants Gender: M = 26/F = 19 Age: 43.1 ± 10.5 years **Health Control:** 35 participants Gender: M = 17/F = 18 Age: 43.4 ± 14.5 years	**Chronic Tinnitus:** Duration: 5.26 ± 4.1 years TQ score: 43.2 ± 9.6 **Recent Tinnitus:** Duration: 8.1 ± 1.6 weeks TQ score: 37.3 ± 15.8	**Chronic Tinnitus:** HNMT **Recent Tinnitus:** HNMT **Health Control:** No intervention	• Visual Continuous Performance Task	• Negative correlation between tinnitus duration and task error rates was suggested at baseline. • Findings suggested treatment helps shift the attention from tinnitus to cognitive tasks in patient with chronic tinnitus.
Krings et al. (2015)	• Whether D-cycloserine facilitate the effects of BFP on tinnitus and cognitions	**Total: 34 participants** **Study group:** 17 participants Gender: M = 9/F = 8 Age: 59 (49–63) **median (range)** **Control group:** 17 participants Gender: M = 11/F = 6 Age: 55 (37–63) **median (range)**	**Study group:** Duration: 4.8 (1–37) TFI score: 47 (10–76) **Control group:** Duration: 10.0 (5–57) TFI score: 44 (14–65)**In median (range)**	**Study group:** BFP with D-cycloserine **Control group:** BFP with placebo	• Stroop Color and Word Test • Paced Auditory Serial Addition Test • CFQ	• There was a reduction on TFI scores and improvement in cognitive tests after intervention compared with baseline. • However, only significant differences between study groups were suggested in CFQ.
Kröner-Herwig et al. (2006)	• Whether patient variables can predict the therapy outcome	**Total: 56 participants** Gender: M = 35/F = 21 Age: 53.65 ± 11.36 years **Condition 1:** 27 participants **Condition 2:** 29 participants	**Total sample:** Duration: 5.57 ± 5.22 years TQ score: 13.35 ± 11.51 **Score difference post intervention**	**Condition 1:** Cognitive Behavioral Tinnitus Coping Training (TCT) **Condition 2:** Habituation-Based Training (HBT)	• Tinnitus-Dysfunctional-Cognitions questionnaire	• No correlation between dysfunctional cognitions and therapy outcome were suggested at post-treatment. • A small negative correlation was found but did not reach significant level.

*BFP, Brain Fitness Program; CFQ, Cognitive Failure Questionnaire; DF, Discussion forum; FDT, Frequency Discrimination Training; GCBT, Group Cognitive Behavior Therapy; HT, Habituation Based Training; HNMT, Heidelberg Neuro-Music Therapy; ICBT, Internet Cognitive Behavior Therapy; TCT, Tinnitus Coping Training; TFI, Tinnitus Function Index; THI, Tinnitus Handicap Inventory; TRQ, Tinnitus Reaction Questionnaire; THQ, Tinnitus Handicap Questionnaire; TQ, Tinnitus Questionnaire*.

Five different self-reported tinnitus assessment scales were used to measure tinnitus severity. They were the Tinnitus Handicap Inventory (THI) (Hesser et al., 2009; Conrad et al., 2015; Kallogjeri et al., 2017), Tinnitus Questionnaire (TQ) (Hiller and Haerkötter, 2005; Kröner-Herwig et al., 2006; Krick et al., 2017), Tinnitus Reaction Questionnaire (Hesser et al., 2009); Tinnitus Handicap Questionnaire (THQ) (Hoare et al., 2014), and Tinnitus Functional Index (TFI) (Krings et al., 2015). It is noteworthy that Kröner-Herwig et al. (2006) reported the changes in the TQ scores postintervention instead of the score at baseline and postintervention. Even though different measures of tinnitus severity were used, the majority of participants was identified as having moderate tinnitus severity.

A number of different interventions were used in these studies, i.e., cognitive behavioral therapy (Hiller and Haerkötter, 2005; Kröner-Herwig et al., 2006; Conrad et al., 2015), brain fitness program (Krings et al., 2015; Kallogjeri et al., 2017), sound therapy (Hesser et al., 2009; Krick et al., 2017), and frequency discrimination training (Hoare et al., 2014). Of these, the brain fitness program was designed specifically for tinnitus sufferers to facilitate neuroplasticity for preservation and improvement in cognitive ability.

In the 8 studies, 11 different evaluations of cognitive functions were used. Three studies used self-reporting questionnaires as outcome measures (Hiller and Haerkötter, 2005; Kröner-Herwig et al., 2006; Conrad et al., 2015). The other five studies used behavioral measurements with two using combinations of different tests to evaluate different aspects of cognitive functions (Krings et al., 2015; Kallogjeri et al., 2017), and three studies using three different behavioral tests to evaluate cognitive functions (Hesser et al., 2009; Hoare et al., 2014; Krick et al., 2017).

### Risk of Bias Within Studies

**Figure 2:**
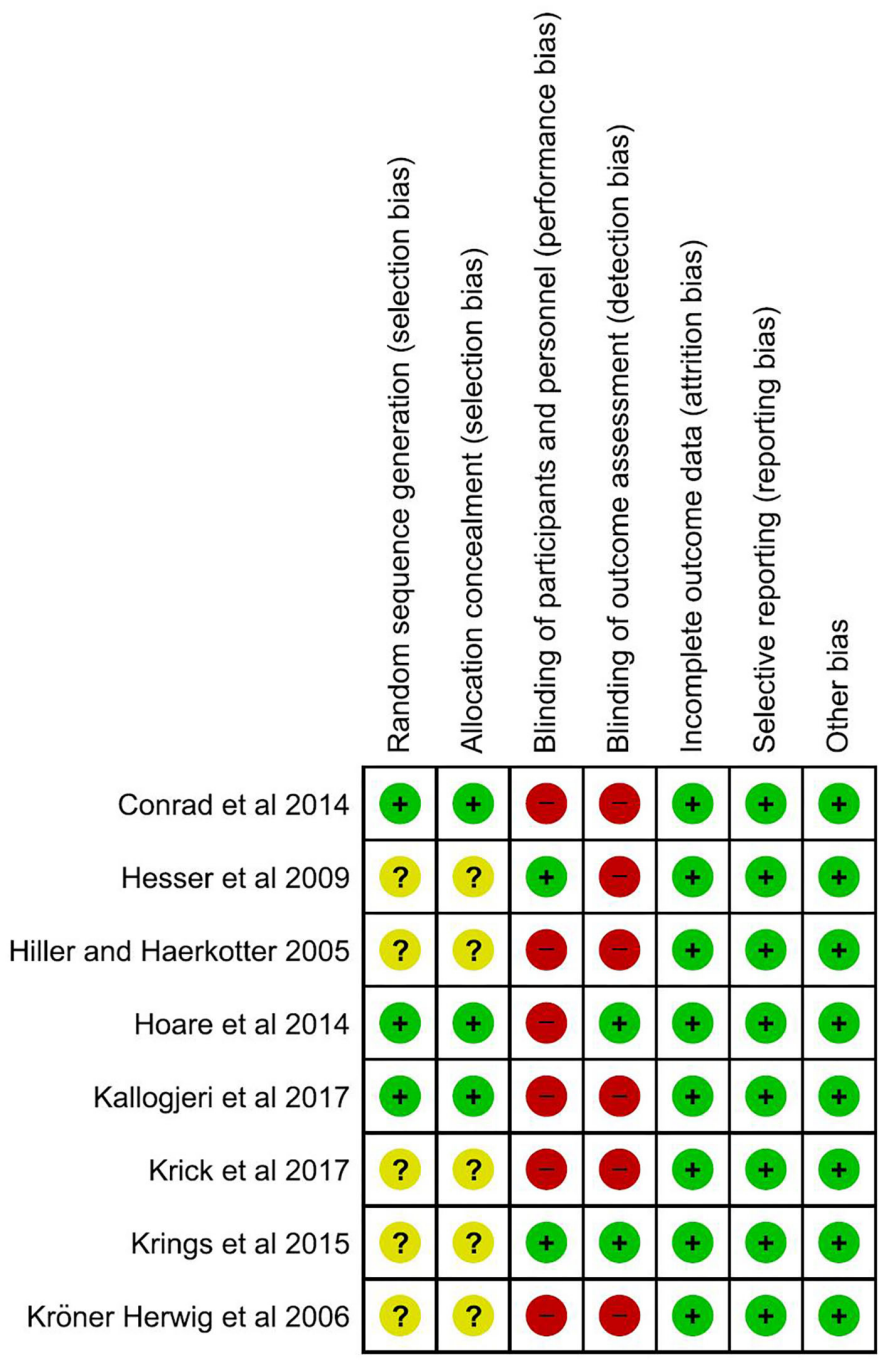
Risk of bias assessment of the included studies.

Figure 2 shows the methodological quality of the eight studies. A small proportion and similar dropout rate occurred among the intervention and control groups in almost all studies, and consequently, bias due to attrition was low in all studies. Additionally, outcomes stated to be measured were all reported across studies, leading to a low risk of reporting bias. However, caution is required when interpreting the results of this review, as there is a possibility that the results might be influenced by publication bias. Although all studies were designed as randomized control trials, most studies did not provide details as to how the randomization and allocation concealment was conducted. As a result, a moderate risk of bias in randomized allocation was found in most studies. Of these included studies, Hoare et al. (2014) and Krings et al. (2015) provided sufficient information to avoid the risk of bias in most domains and provided a high-quality methodology. Although most studies did not report on the blinding of participants and outcome assessors, it is understandable that blinding might not be possible as the use of a placebo in the control group could be difficult in these types of studies, especially in those using no intervention group as comparison.

Because the sample size differs across the included studies (the participant numbers varied from 34 to 128), GPower 3.1 was used for conducting power analysis by extracting the sample size, mean differences between the groups, standard deviation, and alpha level from the included studies. Of these studies, five studies provided sufficient information for power analysis (i.e., Hiller and Haerkötter, 2005; Hesser et al., 2009; Hoare et al., 2014; Conrad et al., 2015; Krick et al., 2017). The study by Krick had the greatest power of over 80%, and Conrad's study provide a medium power of around 55%. However, the three remaining studies (Hiller and Haerkötter, 2005; Hesser et al., 2009; Hoare et al., 2014) could be considered underpowered as they only had the power of 10, 30, and 15%, respectively. For example, a couple of studies demonstrated significant differences in both self-reported assessments of tinnitus and cognitive performance tests between the tinnitus patient group and the control group. Krick et al. (2017) included 113 participants (statistical power > 80%), whereas Hesser et al. (2009) had a small sample (18 participants in the study group vs. 17 controls, 35 participants in total, estimated statistical power = 10%) that the age differed significantly between patient group and control group. Therefore, their significance found by Hesser et al. (2009) should be interpreted with caution due to the possible age influence on the measurements. In contrast, the power analysis was unable to be conducted for the other three studies (i.e., Kröner-Herwig et al., 2006; Krings et al., 2015; Kallogjeri et al., 2017) because they did not provide essential information to calculate the mean differences between groups for power analysis. Therefore, it would be important for these studies to provide sufficient information on the power calculation, which would enable to improve the research efficiency and reliability of the research findings.

### Impact of Tinnitus Interventions on Cognitive Functions

Hiller and Haerkötter (2005) and Conrad et al. (2015) both reported a positive impact of intervention on tinnitus perception and cognitive function. As shown in Table 1, their results using CBT showed significant improvements in tinnitus-related distress and cognitive dysfunction in comparison to control groups. In addition, the effect was long-term determined from the follow-up period. It should be noted that only self-reporting outcome measures were used in both studies, with no evidence gained from subjective behavior performance tests.

The brain fitness program and the program in combination with the antianxiety drug of D-cycloserine (DCS) used by Krings et al. (2015) and Kallogjeri et al. (2017), respectively, improved the performance in behavioral cognitive tests, but no significant difference was found between the tinnitus and control groups. In addition, the improvement in memory and attention assessed using a self-reporting questionnaire was found in the tinnitus group in comparison to the controls. However, it should be noted that the results of the study by Kallogjeri et al. (2017) might be affected by the nonblinding of participants because this study used nonintervention as the control group. The intervention effects may be characterized as a possible response bias because of different perspectives between intervention and nonintervention groups.

In the study by Hesser et al. (2009), the effect of background sounds on tinnitus intrusiveness, and cognitive function was compared. The group with control of background sounds chose a sound and loudness level they preferred, while participants in the second group had no control of background sounds and had to listen to a sound, which was determined for them in type and loudness over the trials. The results showed that participants in the group with controlled sound showed a significantly worse self-rated tinnitus interference over the trials, together with poorer performance in the Digit-Symbol Substitution Test than those receiving no control of background noise. This seems an unexpected result because a positive effect on reducing tinnitus perception was found when a masking noise at the same loudness was used (Vernon and Meikle, 1981; Aytac et al., 2017). The result was interpreted as indicating that controlled masking sounds may have a positive effect on the perception of tinnitus interference initially. However, the participants may increasingly experience that controlled masking sounds eventually lead to increased tinnitus interference (Hesser et al., 2009). An early study also found that excessive use of sound to mask tinnitus was associated with increased tinnitus severity and emotional distress in a sample of individuals with tinnitus (Budd and Pugh, 1996).

On the other hand, Jones and Macken (1993) suggested that complex auditory stimuli had a negative impact on cognitive processing when they investigated the effect of irrelevant tones on working memory. Because tinnitus often varies in pitch and loudness (Andersson et al., 2002), it would be interesting to know the association between tinnitus interference and cognitive processing function assessed using the Digit-Symbol Substitution Test. However, the study by Hesser et al. (2009) did not examine whether increased tinnitus interference was correlated with poorer cognitive performance.

Krick et al. (2017) investigated the effect of tinnitus duration and Heidelberg Neuro-Music therapy on visual attention in a total of 113 participants. This therapeutic approach is a manualized short-term music intervention, consisting of four components, and they are counseling, resonance training, neuroauditive cortex training, and tinnitus reconditioning (Argstatter et al., 2012). A negative correlation was found between tinnitus duration and visual continuous performance task error rates. This suggests that participants with chronic tinnitus make fewer mistakes in visual continuous performance tasks and suggests that Heidelberg Neuro-Music therapy helps shift attention from the tinnitus to cognitive tasks in patients with chronic tinnitus when compared to those patients with recent-onset tinnitus (Krick et al., 2017).

Hoare et al. (2014) evaluated the tinnitus severity and cognitive function of sustained attention following frequency discrimination training (FDT). The results suggested that FDT did not have a positive impact on tinnitus severity measured using THQ in the traditional FDT group and the computer-gameplay-based FDT group. In addition, no significant changes in The Test of Everyday Attention were found in any groups in this study. The negative results are likely due to only one cognitive function measure (i.e., Test of Everyday Attention) being used to assess sustained attention. The Test of Everyday Attention might not sensitively identify the changes in cognitive performance of attention as a result of FDT intervention. A review by Shipstead et al. (2012) suggests that changes in cognitive performance should be assessed using various tasks. This would improve the accuracy of detecting any changes in cognitive performance.

### Cognitive Functions as a Predictor of Intervention Outcomes

Among all the included studies, only two studies addressed whether cognitive dysfunctions would predict poorer intervention outcomes. Conrad et al. (2015) examined cognitive dysfunction as a possible predictor of the effectiveness of CBT on tinnitus-related distress. No association was found between cognitive dysfunction and therapy outcome when using the complete sample. There was, however, after separating the sample into ICBT and GCBT groups, a negative correlation between catastrophic tinnitus-related thoughts and outcome for ICBT, but not for GCBT, i.e., a higher score of subscales at baseline predicted poorer therapy outcomes in emotional tinnitus-related distress in ICBT. In the follow-up assessments, no correlations were observed, suggesting that the negative impact of catastrophic thoughts did not have any long-term effect on therapy outcomes. However, inconsistent results were found in the study by Kröner-Herwig et al. (2006). Although they hypothesized that a higher level of catastrophic tinnitus-related cognition results in a poorer intervention outcome, their results did not suggest any negative association between dysfunctional cognitions and therapy outcomes. The discrepancy between these studies may be caused by the use of different cognitive assessments (i.e., Tinnitus Cognitions Scale and Tinnitus-Dysfunctional-Cognitions Questionnaire in Conrad's study and Kröner-Herwig's study, respectively) and different outcome measures of tinnitus severity (i.e., TQ in Kröner-Herwig's study vs. THI in Conrad' study). In addition, a weak significant correlation (*p* = 0.045) between self-reported questionnaire of cognition and intervention outcomes found in Conrad et al. (2015) may be due to increase in the sample size, because Conrad et al. (2015) had a larger sample size than Kröner-Herwig et al. (2006) (128 vs. 58 participants).

## Discussion

This systematic review aimed to investigate the association between the effectiveness of tinnitus interventions and cognitive functions in patients with tinnitus by critically analyzing relevant randomized control trials. Most of the included trials utilized behavior therapies as tinnitus interventions with a focus on cognitive training. The results suggest that interventions used in these trials not only facilitate tinnitus management but also improve cognitive functions measured using either self-reported questionnaires or behavior performance tests in patients with tinnitus.

CBT, a psychological treatment that focuses on cognition and emotion, provides a better understanding of the relationships between our feelings, thinking, behaviors, and environment, and the ways in which these can become problematic. Currently, there is substantial evidence on the positive benefits of CBT on various mental health conditions and chronic psychological disorders as well as patients with chronic tinnitus (Hesser et al., 2011; Hans and Hiller, 2013). As a result, the National Institute for Health and Care Excellence (NICE) on tinnitus assessment and management recommends CBT as an effective tool to reduce the tinnitus-related distress, and thus alleviate the severity of tinnitus.

In this review, cognitive behavioral therapy and brain fitness program were used in four included studies, primarily focusing on alteration of cognition and emotion (Hiller and Haerkötter, 2005; Conrad et al., 2015; Krings et al., 2015; Kallogjeri et al., 2017). Their results showed positive outcomes in terms of improvement in tinnitus severity and cognitive function, which indicates that improvement in cognitive ability and alteration in how tinnitus and its consequences are perceived leads to positive attitudes toward tinnitus and reduction in tinnitus severity. Interventions that focus on cognition and emotion were also used in studies of other disciplines of chronic pain, and study results suggested that improved cognitive ability and positive emotions have an effect on symptoms alleviation (George et al., 2010; Keefe and Somers, 2010). This suggests that psychological factors, e.g., cognition, emotion, and behavior play an important role for patients with a chronic disease to cope with chronic symptoms, such as tinnitus.

A previous study by Andersson and McKenna (2006) suggested that cognitive deficits, cognitive bias, and beliefs and attitudes toward tinnitus are three important stages on the course from tinnitus perception to annoyance. Of these stages, cognitive deficits in terms of control of attention lead to disruption of information processing when tinnitus competes with other activities. Evidence suggested that patients with tinnitus performed worse in the cognitive tests of attention compared with healthy control without tinnitus (Jackson et al., 2014; Cardon et al., 2019). A cognitive bias in patients with tinnitus can be defined as a systematic error, particularly in memory and attention, when they are processing and interpreting information that occurs around them and thus affects judgement and decision-making. Evidence suggested that patients with tinnitus paid selective attention to information related to tinnitus and tend to make decisions based on a biased recourse (Andersson et al., 2000, 2003). Beliefs and attitudes toward tinnitus are also important in terms of moderating the adverse effect of tinnitus. The study by Conrad et al. (2015) showed that negative thoughts of tinnitus were negatively associated with the effect of ICBT on tinnitus distress.

According to the model proposed by these authors, it is not sufficient for tinnitus to attract attention for it to be annoying, and even though tinnitus is correlated with psychopathology, it does not always lead to distress. Tinnitus is likely to become a serious problem when it interferes with the ability of the patient to think, when his or her general attitude in life is either anxious or depressive and when tinnitus is seen as the cause of the problems (e.g., insomnia and concentration problems). A meta-analysis by Hesser et al. (2011) concluded that CBT has a positive impact on annoyance and distress associated with tinnitus. Therefore, with an understanding of underlying issues, together with a focus on retraining thoughts and alteration of behaviors, it is possible that therapeutic interventions on cognitive functions in terms of making changes to how people feel can provide a viable and effective approach in ameliorating tinnitus.

It is noteworthy that sound-based interventions demonstrate positive results in both tinnitus and cognitions in the study by Krick et al. (2017). This suggests that better performance in cognitive function is the result of less attention being placed on the tinnitus and indicates an association between the benefits of sound-based approaches and better cognitive function in patients with tinnitus. However, more studies using standard intervention and outcome measures need to be undertaken to reach a solid conclusion of the association between the effectiveness of sound-based interventions and cognitive function in patients with tinnitus. As discussed earlier, although the study by Conrad et al. (2015) found a negative correlation between catastrophic tinnitus-related thoughts (a form of cognitive dysfunction) and therapeutic outcomes on emotional changes, it remains unclear whether cognitive functions would be a predictor of outcomes when using tinnitus interventions.

However, negative correlations between cognition and treatment outcomes were reported in other disciplines, such as chronic pain. The cognitive dysfunction is consistently correlated with reported pain severity and poor treatment outcomes in patients with rheumatic diseases (Edwards et al., 2011). The authors suggested that negative attitudes and beliefs related to pain can be used as a predictor for non-improvement after surgery or other treatments. A similar conclusion has been drawn by Quartana et al. (2009), who conclude that catastrophizing thoughts toward the chronic pain can lead to more difficulties in suppressing pain-related thoughts and associate with a number of important outcomes in pain treatment.

Various outcome measures of cognitive functions have been used in categorizing either self-reported measurement or behavioral cognitive tests. Evidence shows a lack of correlation between self-reported measures and performance in cognitive function tests. For example, a study by Hallam et al. (2004) examined the correlation between cognitive performance and score in a self-reported CFQ. The results showed that CFQ was associated with performance on the dual-task version delayed serial digit recall (a working memory test), but the correlation coefficients were low (between 0.21 and 0.33). Among the included studies within the present review, only two used both self-reported measurement and behavioral tests to measure cognitive functions. The study by Krings et al. (2015) revealed improvement in tinnitus, behavioral, and self-reported cognitive functions, while Kallogjeri et al. (2017) only reported improvement in tinnitus and self-reported cognitive functions. Therefore, the reliability of self-reported measurement or behavioral cognitive tests needs to be further examined.

Furthermore, although confounding factors, e.g., age, degree of hearing loss, depression, and anxiety, have been considered and controlled in most of the individual studies, the average age of participants ranged between 45 and 55 years old. A psychological study testing memory capacity among people aged from 50 to 79 suggested that the precision of the evaluations of individuals' own abilities is dramatically altered by age-related changes in self-regard and lifestyle (Rabbitt and Abson, 1991). Moreover, a cross-sectional and longitudinal study by Wielgos et al. (1999) also showed a significant negative correlation between the cognitive performance test, digit symbol task, and age. Therefore, it would be useful to consider a wider age range in future studies on the association between the effectiveness of tinnitus interventions and cognitive functions in patients with tinnitus.

Evidence indicates that tinnitus is commonly associated with hearing loss (Baguley et al., 2013; Basso et al., 2020). Because several previous studies suggest the associations between hearing loss and impairment of cognitive function (e.g., memory, executive function, and attention) (Lin, 2011; Saunders et al., 2018), it is therefore important to consider the negative influence of hearing loss on cognitive performance when investigating the impact of tinnitus on the cognitive function in individuals with tinnitus. Waechter et al. (2020) found that working memory was not influenced by tinnitus but rather by hearing status in patients with tinnitus. Therefore, the factor of hearing loss should be considered when the studies compare cognitive performance in individuals with tinnitus and without tinnitus.

One of the selected studies recruited patients with chronic and recent-onset tinnitus where there was no difference in tinnitus severity but poorer performance in cognitive tasks in patient with chronic tinnitus after intervention (Krick et al., 2017). This is consistent with the findings by Trevis et al. (2016), which investigated a variety of components that affect attention switching, including cognitive control, inhibition, and working memory in patients with tinnitus. Their results suggested that major impairment in cognitive control and inhibition as well as lower emotional well-being can be caused by long-lasting tinnitus. The difficulties in attention switching caused by the long-term chronic tinnitus may be associated with the structural change between attention-related and self-reference brain networks (Lanting et al., 2016; Leaver et al., 2016). Therefore, there is a need for more evidence on the influence of tinnitus duration on the association between the effectiveness of tinnitus interventions and cognitive functions in patients with tinnitus.

The present review does need to be considered in light of several limitations. Although the quality of each included study appears appropriate, due to some heterogeneity in tinnitus interventions and behavioral cognitive measurements, it is difficult to conduct a meta-analysis and consequently did not provide a quantitative review of the effect of tinnitus intervention on both tinnitus severity and cognitive performance. Therefore, a more homogeneous set of interventions and outcome measurements is needed in order to be conclusive as to the association between tinnitus interventions and cognitive functions in patients with tinnitus.

## Conclusion

Favorable results in both tinnitus severity and cognitive functions of patients with tinnitus were reported in most of the included studies in this review. However, whether cognition can predict treatment outcomes is unclear due to insufficient evidence. The significant heterogeneity of interventions and outcome measures in the included studies makes it challenging to compare the studies' outcomes. Therefore, the association between tinnitus interventions and cognitive functions in a patient with tinnitus should be interpreted with caution. In addition, self-reported measures and behavioral assessments in cognition should both be employed in future trials. The influence of age and tinnitus duration should be considered as well. Future research with a more homogeneous methodology focused on the effect of sound-based interventions on both tinnitus and cognitive functions and whether cognition is a predictor of treatment outcome is needed.

## Data Availability Statement

The original contributions presented in the study are included in the article/supplementary materials, further inquiries can be directed to the corresponding author/s.

## Author Contributions

TL, NP, and FZ conceived and designed the review. TL and ZC developed search strategies and conducted database search and article selection. All authors contributed to the writing.

## Conflict of Interest

The authors declare that the research was conducted in the absence of any commercial or financial relationships that could be construed as a potential conflict of interest.
